# Comparison of tolerance to sunlight between spatially distant and genetically different strains of *Lymantria dispar* nucleopolyhedrovirus

**DOI:** 10.1371/journal.pone.0189992

**Published:** 2017-12-20

**Authors:** Yuriy B. Akhanaev, Irina A. Belousova, Nikita I. Ershov, Madoka Nakai, Vyacheslav V. Martemyanov, Viktor V. Glupov

**Affiliations:** 1 Laboratory of Insect Pathology, Institute of Systematics and Ecology of Animals SB RAS, Novosibirsk, Russia; 2 Laboratory of ecological parasitology, Institute of Systematics and Ecology of Animals SB RAS, Novosibirsk, Russia; 3 Institute of Biology, Irkutsk State University, Irkutsk, Russia; 4 Molecular Genetics Department, Institute of Cytology and Genetics SB RAS, Novosibirsk, Russia; 5 Institute of Agriculture, Tokyo University of Agriculture and Technology, Fuchu, Tokyo, Japan; 6 Biological Institute, National Research Tomsk State University, Tomsk, Russia; INRA, FRANCE

## Abstract

Baculoviruses are a family of insect-specific pathogenic viruses can persist outside for long periods through the formation of occlusion bodies. In spite of this ability, the UV of sunlight is an essential factor that limits the survival of baculoviruses outside the host. In the current study, we compared the UV tolerance of two strains of *Lymantria dispar multiple nucleopolyhedrovirus* (*LdMNPV*), which were isolated in spatially different regions (*LdMNPV*-27/0 in Western Siberia (Russia) and *LdMNPV*-45/0 in North America (USA)) and dramatically differ in their potency. We exposed the studied strains to sunlight in an open area for 0.25, 0.5, 1, and 2 hours and later perorally inoculated host larvae with the same doses of virus (5x10^5^) and with doses leading to same effect (LD_90_). We observed that strain *LdMNPV*-45/0, which previously showed high virulence against *L*. *dispar* larvae, was more sensitive to UV irradiation (estimated as the relative rate of inactivation (*r*, h ^-1^) and as the half-life of the virus (τ_1/2,_ h)) compared to *LdMNPV*-27/0. Exposure to sunlight induced a significant delay of *LdMNPV*-45/0-induced pathogenesis already after 0.25 h of sunlight exposure, while for *LdMNPV*-27/0 this delay was occurred only after 2 h exposure in spite of used concentrations. We also compared the sequences of the main structural proteins of the studied strains as UV light contributes not only to genome damage in viruses but also to structural protein damage. The most prominent genetic difference between the structural proteins of the strains was related to the loss of the virus enhancin factor-1 (*vef-1*) gene in the *LdMNPV*-27/0 strain. Thus initially highly potent viral strain (such as *LdMNPV*-45/0) is not recommend to use in the regions (or forest stand density) with high UV load. The role of virus enhancin factor-1 in baculovirus tolerance to UV needs for following studies.

## Introduction

Baculoviruses are a unique family of viruses that exclusively infect insects (according to International Committee of Taxonomy of Viruses) and often lead to mass epizootics in host populations [1–2]. Baculoviruses penetrate the host organism perorally, and are then colonized the host organism and lead to the death of the host at juvenile stages; this is followed by liquefaction of the cadavers. The last event leads to the contamination of the surrounding surfaces (including the leaf surface) and the horizontal transmission of the virus to healthy individuals [3–4]. Baculoviruses are able to persist outside the host for long periods due to the formation of polyhedra/occlusion bodies, which protect virions from the effects of environmental factors, including UV light [5]. The genes encoding polyhedrin/granulin proteins, major structural components of occlusion bodies, are strongly conserved and are used as a molecular criterion for baculovirus taxonomy [6]. Another feature of baculoviruses (inherent to many other viruses) is the ability to infect the host without a symptomatic appearance [7–13]. During asymptomatic infection, a baculovirus can be transmitted from parents to progeny and is activated by the effects of stress factors on the host organism, leading to its death [14].

*Lymantria dispar* L. (Lepidoptera: Erebidae) is a widely distributed forest defoliator that originally inhabits the temperate forests of Asia, Europe, and Northern Africa [15–18]. At the end of the nineteenth century, the European strain of *L*. *dispar* was introduced into the eastern part of North America, where it continues to successfully adapt and produce regular outbreaks, thus expanding the area of infestation in a westerly direction. *L*. *dispar* is the host for one of the representatives of Baculovirus, namely *Lymantria dispar multiple nucleopolyhedrovirus* (*LdMNPV*). *LdMNPV* often induces spontaneous epizootics in *L*. *dispar* populations in many parts of the host area and is used by biologists in many countries to develop *LdMNPV* products for *L*. *dispar* population management [19–21].

Because of the wide distribution of *L*. *dispar* populations in the northern hemisphere, insects inhabit areas with significantly different climate types (from marine to continental) [22]. Consequently, the effects of environmental factors on the host (*L*. *dispar*) and its obligate parasite (*LdMNPV*) significantly differ. One essential factors limiting the survival of the virus outside is UV radiation from sunlight [23]. UV light is actively adsorbed by DNA molecules and induces the formation of pyrimidine dimers in DNA chains [24–26]. In spite of the ability of baculoviruses to form crystalline polyhedrin or granulin (which provides protection from environmental factors, including UV light), direct irradiation with the UV spectra of sunlight significantly decreases the viability of baculoviruses [27–30]. UV-A (315–400 nm) and UV-B (280–315 nm) are the most important parts of UV sunlight spectra because of their ability to penetrate the ozone layer and reach the earth’s surface, while waves shorter than 295 nm (UV-C and partially UV-B) are absorbed by the ozone layer [31]. The intensity of UV on the earth’s surface is dependent not only on remoteness from the sun (i.e., latitude, season) but also the cloudiness in the atmosphere, which is closely related to the dominance of cyclones or anticyclones [26, 32–33].

We recently found that three strains of *LdMNPV* isolated in Northern American populations of *L*. *dispar* possess more than tenfold higher potency over three *LdMNPV* strains isolated in a continental Asian (Western Siberia) *L*. *dispar* population [34]. The potency of the American strains was not dependent on the host population, as was shown in the cross bioassay. Moreover, the genome of one representative American strain (*LdMNPV*-45/0) was significantly different from the representative Asian strains (*LdMNPV*-27/0) [34–35]. The aim of the current work was to compare the tolerance of the studied strains (*LdMNPV*-27/0 and *LdMNPV*-45/0), which were isolated in different climate zones, to the effects of UV sunlight radiation.

## Materials and methods

### Insects and nucleopolyhedrovirus

Egg masses of diapausing insects were collected in the Novosibirsk region (Western Siberia 54.33^o^N 81.13^o^E) in autumn 2015 and were kept in a refrigerator at +4°C. The population of *L*. *dispar* was at the raising phase of population dynamics. The progeny of 100 females were mixed and used as a single stock for randomized assignment of insects to treatment. In the spring, insects were reared under laboratory conditions at permanent room temperature (+23°C) and in a natural daylight regime. To prevent surface contamination of the eggs by external NPVs, we used sodium hypochlorite for sterilization, followed by rinsing in sterile water [36]. Before the experiment, insects were reared in plastic containers (100 individuals/20 l container) and were fed cut branches of silver birch *Betula pendula* Roth. The branches were preliminarily washed using sterile water to exclude the presence of *LdMNPV* on the leaves. Before larvae rearing, a pool of randomly chosen eggs was taken from the stock of egg masses to determine the prevalence of covert *LdMNPV* by PCR (see below).

For insect challenge, we used two strains of *LdMNPV* stored in the collection of the Laboratory of Insect Pathology of the Institute of Systematics and Ecology of Animals SB RAS: strain 45/0 (isolated in the state of Massachusetts, USA (42.15°N; 72.49°W) and kindly given by Dr. John Podgwaite, USDA Forest Service) and strain 27/0 (isolated in the Novosibirsk region, Russia, 55.14^o^N; 75.52^o^E). Both strains were passaged via host larvae at the same time before the study to control the effect of strain storage. Occlusion bodies (OBs) after passaging were isolated from larvae cadavers in the same manner (crushing and following filtering of the suspension), as it is known that varying presence of cuticle debris may affect the UV tolerance of NPVs [37–38]. The strains were not clones and both strains were a mixture of genotypes [34–35].

### Experimental design

The level of tolerance of viral strains to UV sunlight was measured using different durations of exposure for the virus placed on a leaf surface in direct sunlight. To estimate the level of tolerance of the compared strains to UV, we used four exposure times: 0.25, 0.5, 1, and 2 hours. The exposure treatments were performed in the second half of May at noon in clear weather. To minimize the effects of the differences in the power of UV radiation associated with time of day, all exposure treatments were started at the same time. The power of UV radiation during the exposure period varied between 2.5 and 2.7 mW/cm^2^. We used two methods of comparison for applied virus: comparison of equal doses and of equal effects (i.e., virus-induced mortality), as the strains used significantly differed in potency. The slope of dose-effect dependence was the same for both studied strains [34].

### Challenging insects with *LdMNPV* strains

**Fig 1:**
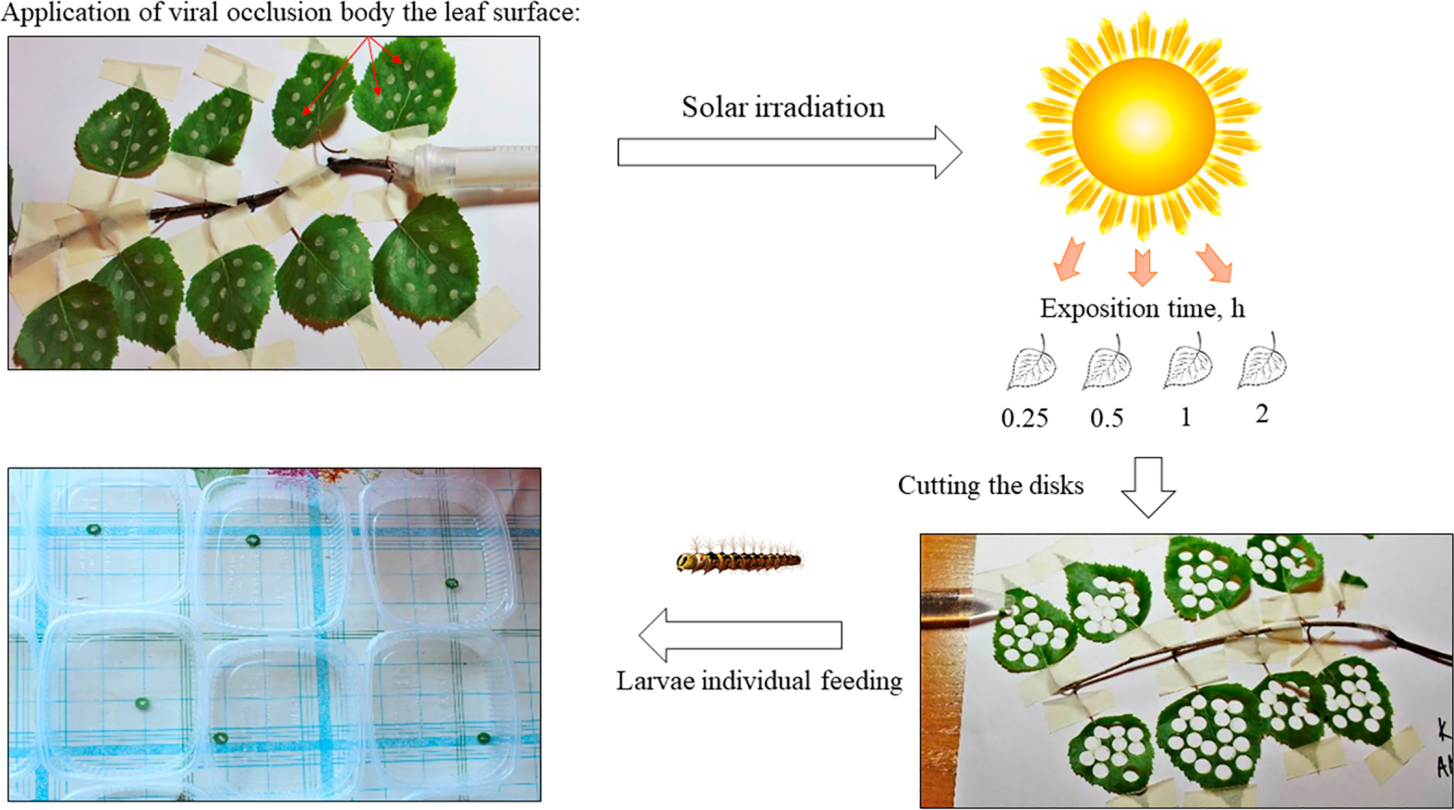
Scheme for UV treatments of *LdMNPV*.

For the experiment, we used fourth instar larvae on the second day after molting. For the inoculation we used doses of virus of approximately LD_90_: 5×10^5^ OBs/larvae for *LdMPNV*-45/0 and 5x10^6^ OBs/larvae for *LdMNPV*-27/0. To control the effect of different doses, an additional dose of *LdMNPV*-27/0 with the same dose as *LdMNPV*-45/0 (i.e., 5×10^5^ OBs/larvae) was used. We did not include data for 5x10^6^ OBs/larvae for *LdMNPV*-45/0 in the comparison, as this dose ten-fold exceeded the maximal mortality rate. We also did not measure the potency of the studied viral strains for the current *L*. *dispar* population, as our earlier study clearly showed that potency was determined only by virus features [34]. The virus challenge was performed as follows. The fixing dose of *LdMNPV* OBs, contained in a drop of distilled water (5 μl), was applied to the leaf surface, and the leaves were dried at room temperature. The drop covered the same area of leaves in all treatments (the drop diameter was ca. 4 mm) to control the thickness of the viral layer and avoid differences in the shadow by viral OBs. To maintain leaf turgor, branches were placed in florist’s tubes and fixed to a sheet of paper with sticky tape. The last manipulation allowed all leaves to be in the same plane to direct all virus contaminated spots towards the sunlight at the same angle. When the drops had evaporated, the leaves were exposed to sunlight. After treatment, we cut small leaf discs (diameter of approximately 10 mm), which were individually given to larvae (Fig 1). Only larvae that totally consumed the discs within 12 hours were used for the subsequent bioassay. After inoculation, larvae were reared individually in plastic containers (125 ml). Despite the individual rearing of larvae, we assigned larvae to three nominal replicates in each treatment for the following statistical comparison. In the control, the same manipulations were performed with distilled water. No mortality was recorded in the control. As not all insects consumed whole discs, the sampling numbers varied from 37 to 60 larvae per treatment per dose. Death and the presence of food were monitored daily. The effects of sunlight on the potency of the viral strains were measured as relative rate of inactivation (*r*, h ^-1^) and virus half-life (τ_1/2_, h) based on estimation of the percentage of original activity remaining (% OAR) [39]. We also measured the time to death and the OB production of *LdMNPV*s strains. The time to death is an important parameter of the effectiveness of the viral product during pest management, while viral productivity characterizes the ability of the studied strain to induce repeated mortality in next host generation via exogenous challenge. The etiology of mortality was detected by light microscopy. The productivity of the virus was estimated with light microscopy using a hemocytometer. We also compare the average size of the *LdMNPV* occlusion bodies as the criteria of the strain morphology with the help of a digital camera and AxioVision 4.8 software.

### Detection of *LdMNPV* prevalence in the stock *Lymantria dispar* population

We also determined the prevalence of covert infection to avoid a possible interaction between covert virus and virus used for exogenous challenging, as it is known that exogenously penetrated baculovirus may activate covert baculovirus infection [3]. To estimate the prevalence of covert *LdMNPV* infection in stock host populations, ten samples of *L*. *dispar* eggs (pools of eggs) containing 10 eggs per pool were randomly chosen from the mixed stock of egg masses for PCR analysis. In total, 100 insects were analyzed. Total DNA was extracted using the phenol-chloroform method with some modification. To prevent surface contamination of the eggs for infection prevalence, we used sodium hypochlorite for sterilization, as described above. Next, pools of eggs were mechanically homogenized with a pestle in a lysis solution containing guanidine isothiocyanate (kit C-8879, VECTOR-BEST, Russia). To control for successful DNA extraction, PCR analysis of the 28Sr RNA gene of *L*. *dispar* was used [40]. A fragment of the polyhedrin gene was used as the target sequence for detecting the presence of *LdMNPV* DNA in the analyzed samples. The amplification reactions for the analysis of the gypsy moth NPV polyhedrin gene contained 50 nM of primer 1, 50 nM of primer 2, DNA at 500 ng/25 μl at the final volume, and the reaction mixture HS-qPCR Mix SYBR (Biolabmix). The reaction conditions were 5 min at 95°C and 40 cycles of 30 sec at 95°C and 1 min at 60°C, with a melting curve of 60–95°C. The primers for the gypsy moth NPV polyhedrin gene were LdPH1268-F 5’-GCACTTCCTCAACTCGGTCA-3’ and LdPH1393-R 5’-CGTTTAGTACGCCGGTCCTT-3’ (Primer-BLAST). Viral DNA detection was performed using SYBR Green on a CFX96 (Bio-Rad). It should be noted that the use of this detection method lowered the limit of DNA detection compared to classical detection by ethidium bromide. DNA extracted from the eggs of a gypsy moth population that was free of *LdMNPV* was used as the negative control. DNA extracted from *LdMNPV* was used as the positive control. The product of the PCR reaction was sequenced to confirm the target sequence.

### Analysis of amino acid similarity of *LdMNPV* structural proteins

UV light contributes not only to genome damage in viruses but also to structural protein damage [41–43]. Thus, we compared the sequences of the main structural proteins of the studied strains. Open reading frames (ORFs) were predicted in the genomic sequences for *LdMNPV*-45/0 (GenBank Acc. KU862282.1) and *LdMNPV*-27/0 (GenBank Acc. KY249580) using the GeneMark.hmm web application [44]. The protein translations were pairwise-aligned to each other using the blastp program. Protein functional domains were searched using the HMMER web application against the EMBL-EBI Pfam database (http://pfam.xfam.org/) and the CD-search service against the NCBI Conserved Domains Database (https://www.ncbi.nlm.nih.gov/Structure/cdd/cdd.shtml).

### Statistical analysis

Sun’s model was used to calculate percentage of original activity remaining (%OAR) at each UV-exposure time using JMP10.0.0 (SAS Institute Inc.) [39]. According to the following formula %OAR = 100 x e^(-r x t)^, 95% confidence limits of *r* as relative rate of inactivation (h^-1^) were compared between *LdMNPV*-45/0 and -27/0. The half-life of each isolate was calculated from the following formula: *τ*_1/2_ = ln (2)/*r*, and its 95% confidence limit was also compared to reveal significant differences. The virus productivity data were tested for normality with the Shapiro-Wilk normality test and were statistically compared using factorial ANOVA. The size of the OBs of the initial strains was compared by one-way ANOVA. The time to death of mortality was compared by the Kaplan-Meier test (Log-rank test). We used Statistica 12.0, SigmaPlot 12.5 and GraphPad Prism 5 software.

## Results

PCR detection did not reveal the amplification of *LdMNPV*-specific DNA fragments in any tested samples of *L*. *dispar* eggs. Thus, the studied population of insects was free from endogenous virus or frequency of virus prevalence was extremely low.

**Fig 2:**
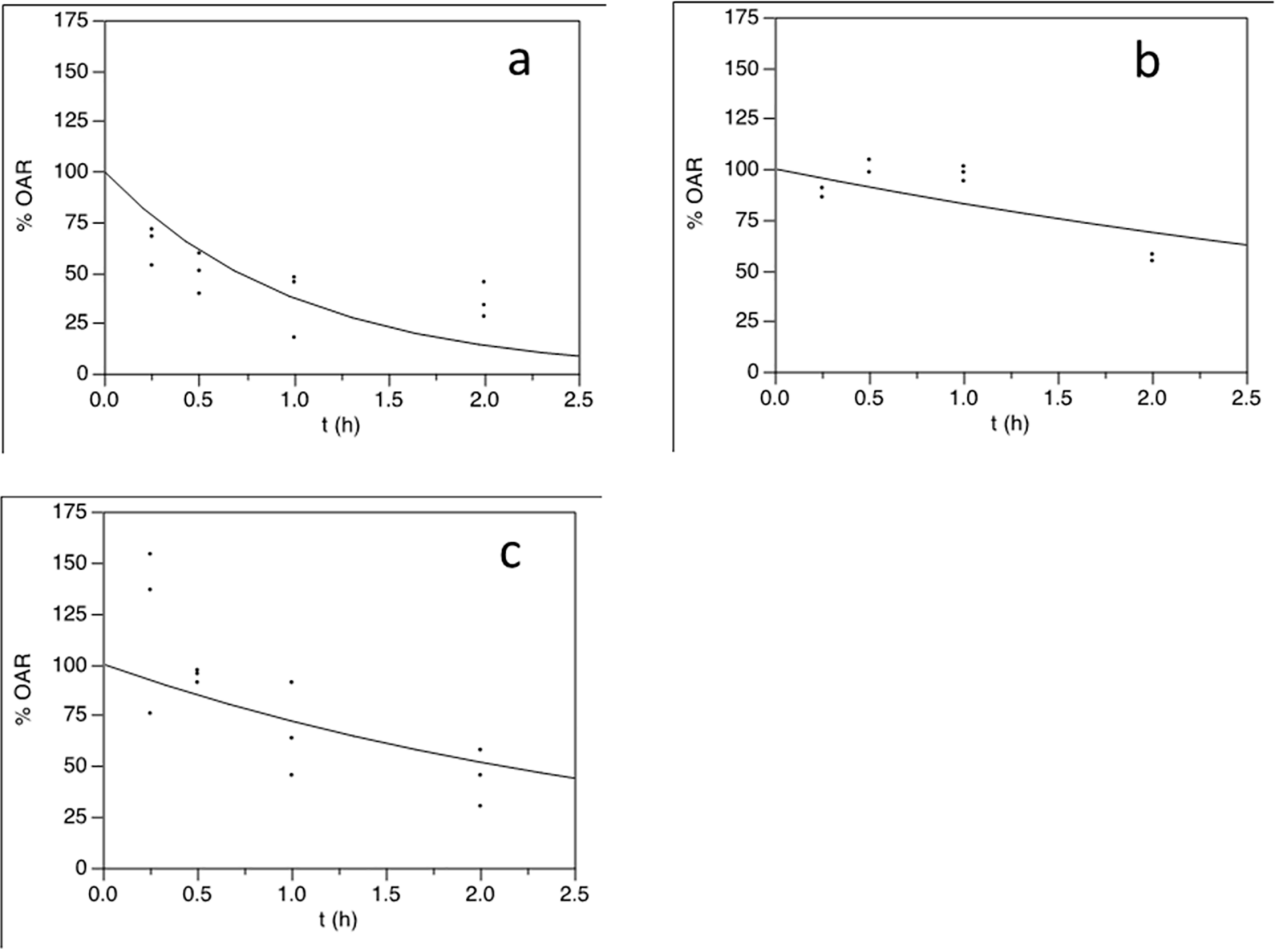
Inhibition of viral potency by sunlight treatment. Percentage of original activity remaining (%OAR) of *LdMNPV*-45/0 (dose 5x10^5^ OBs/larvae, a), *LdMNPV*-27/0 (dose 5x10^6^ OBs/larvae, b) and *LdMNPV*-27/0 (dose 5x10^5^ OBs/larvae, c) strains exposed to sunlight UV.

In an experiment examining the loss of pathogenicity upon irradiation with sunlight, the *LdMNPV*-27/0 retained significantly more potency towards *L*. *dispar* larvae during prolonged periods of UV irradiation (Fig 2), as the relative ratio of inactivation (*r*) was significantly greater for OBs of *LdMNPV*-45/0 than *LdMNPV*-27/0, as observed in the comparison of the “same dose” and “same effect” groups (Table 1). The half-life (*τ*_1/2_) of the *LdMNPV*-27/0 for same impact was longer than for *LdMNPV*-45/0), but a significant difference was only detected for the same impact (LD_90_) (Table 1).

**Table 1:** Inactivation rate and half-life of the *LdMNPV* strains by sunlight.

Strain	dose (OBs/larvae)	*r* (h)*	SE	lower limit	upper limit	*τ*_1/2_ (h)*	SE	lower limit	upper limit
*LdMNPV*- 45/0	5x10^5^ (LD_90_)	0.99^a^	0.15	0.67	1.48	0.70^a^	0.11	0.49	0.92
*LdMNPV* -27/0	5x10^6^ (LD_90_)	0.19^b^	0.04	0.10	0.28	3.71^b^	0.85	2.04	5.37
	5x10^5^ (0.1xLD_90_)	0.33^b^	0.10	0.14	0.58	2.11^ab^	0.66	0.81	3.41

* r shows relative rate of inactivation and τ_1/2_ shows half-life of the virus

Different letters mean the significant differences

Compared to *LdMNPV*-27/0, the *LdMNPV*-45/0 strain led to a more rapid death of larvae when applied at the same concentrations; but *LdMNPV*-27/0 killed larvae faster when equal doses (LD_90_) were used (Tables 2 and 3, S1 Fig). However, exposure to sunlight induced a significant delay of *LdMNPV*-45/0-induced pathogenesis after 0.25 h of sunlight exposure, while for *LdMNPV*-27/0, this delay occurred after only 2 h of exposure in spite of the concentration used. The comparison within each sunlight exposure treatment showed no differences between the studied viral strains of the same concentration (except for the no sunlight treatment), while insects were killed faster in all sunlight treatments when they were infected by *LdMNPV*-27/0 at LD_90_ than when they were infected by *LdMNPV* -45/0 at LD_90_ (Tables 2 and 3, S1 Fig).

**Table 2:** Mean and median time to death of *Lymantria dispar* larvae infected by different *LdMNPV* strains after exposure to sunlight.

Strain (OBs/larvae)	Mean (median) lethal time±SE at the exposition of virus under sunlight				
	0h	0.25h	0.5h	1h	2h
*LdMNPV*-45/0 (5x10^5^)	16.80 ±0.779^a^ (16±0.849)	20.69± 1.073^a^ (23 ±4.498)	20.56±0.733^a^ (--±--)	23.17±0.831^a^ (--±--)	21.68±0.737^a^ (--±--)
*LdMNPV*-27/0 (5x10^6^)	13.35±0.475 ^b^ (13±0.478)	16.92±0.877^b^ (14 ±0.474)	14.08±0.713^b^ (12±0.236)	13.25±0.595^b^ (12±0.491)	15.00±0.331^b^ (--±--)
*LdMNPV*-27/0 (5x10^5^)	19.55±0.873 ^b^ (21±3.320)	16.32±0.681^a^ (15 ±2.027)	21.07±0.933^a^ (24±--)	20.15±0.700^a^ (--±--)	25.20±0.918 ^a^ (--±--)

Different letters indicate significant differences between *LdMNPV*-45/0 and *LdMNPV*-27/0 compared within one exposition treatment. Detailed statistics is given in the Table 3. Dashed line indicates that the calculation of value is impossible

**Table 3:** The results of the log-rank statistics (pairwise comparison) for the time to death of *Lymantria dispar* larvae induced by *LdMNPV*.

OBs/larvae	*LdMNPV*-27/0 vs *LdMNPV*-45/0			
	5x10^6^ vs 5x10^5^		5x10^5^ vs 5x10^5^	
hours	ч^2^	P value	ч^2^	P value
0	4.366	0.037	9.473	0.002
0.25	5.375	0.020	1.563	0.211
0.5	33.843	<0.0001	0.539	0.463
1	38.052	<0.0001	1.725	0.189
2	7.166	0.007	0.583	0.445
*LdMNPV*-27/0	5x10^6^/larva		5x10^5^/larva	
hours	ч^2^	P value	ч^2^	P value
0 vs 0.25	3.675	0.055	1.934	0.164
0 vs 0.5	0.354	0.552	0.504	0.478
0 vs 1	0.0873	0.768	2.040	0.153
0 vs 2	14.276	<0.0001	12.181	<0.0001
*LdMNPV*-45/0	5x10^5^/larva	
hours	ч^2^	P value
0 vs 0.25	11.4	<0.0001
0 vs 0.5	21.041	<0.0001
0 vs 1	39.519	<0.0001
0 vs 2	34.083	<0.0001

**Fig 3:**
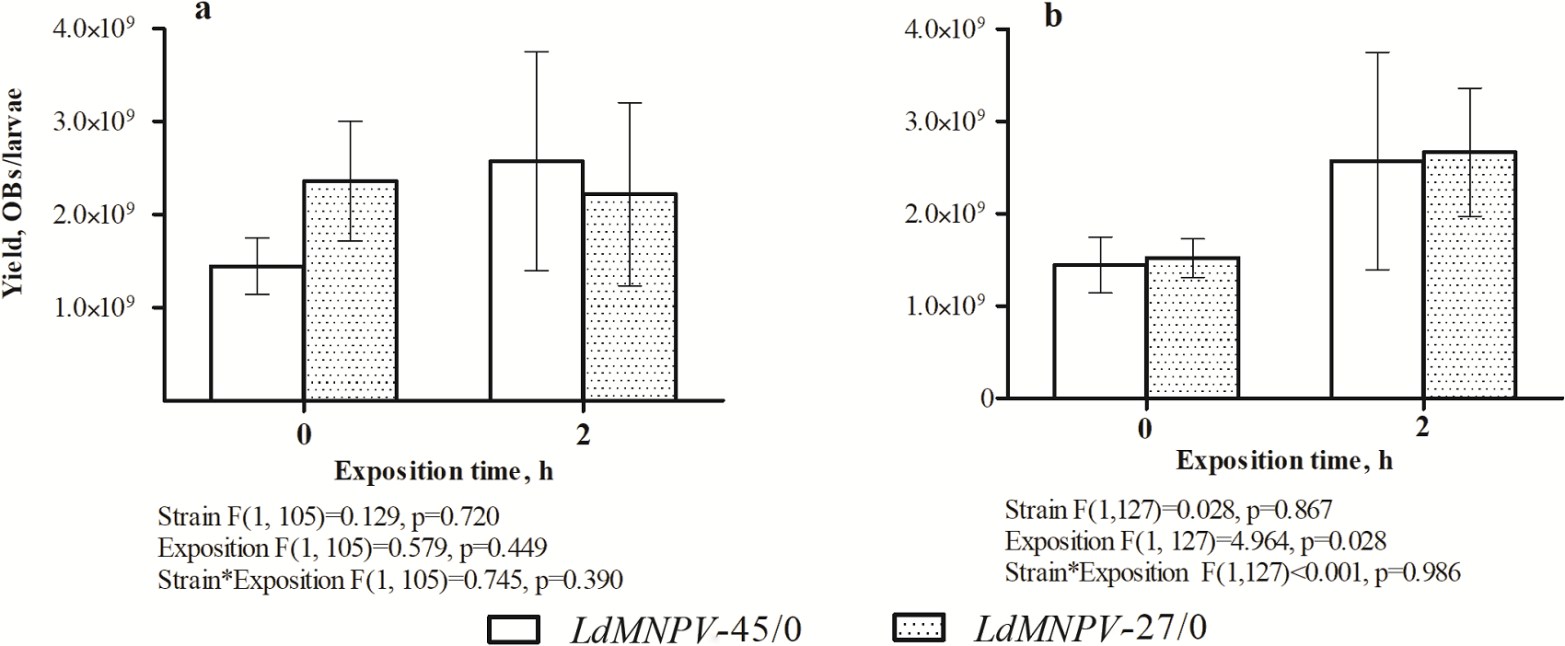
Effect of sunlight treatment on *LdMNPV* productivity. Productivity of *LdMNPV* strains after challenging of *Lymantria dispar* larvae with same dose (5x10^5^ OBs/larvae, a) or a dose leading to the same effect (LD_90_, b) of *LdMNPV* strains. The productivity was calculated using a light microscope with the help of hemocytometer.

The productivity of *LdMNPV*-45/0 and *LdMNPV*-27/0 was not significantly different independently when larvae were treated with the same doses of virus (Fig 3A) and with doses leading to the same effect (LD_90,_ Fig 3B). However, sunlight treatment significantly affected (increased) the productivity of the studied viral strains when strains were compared on the basis of same effect induced (i.e., LD_90,_ Fig 3B). The diameters of the OBs of the initial *LdMNPV* strains used for inoculation were 2.10±0.067 μm for *LdMNPV*-45/0 and 2.06±0.058 μm for *LdMNPV*-27/0, and they were not significantly different (F_1,253_ = 0.028; P = 0.867).

**Fig 4:**
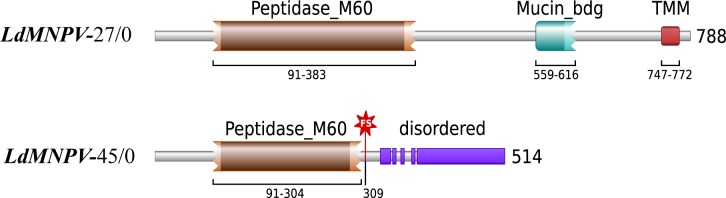
Frameshift in *LdMNPV*-45/0 DNA sequence. A two-nucleotide deletion in the *vef*-2 gene of the *LdMNPV*-45/0 strain resulted in a frameshift (FS) at the 309 amino-acid position, which led to a shortened protein with a partially affected peptidase domain (Peptidase_M60), lost mucin-binding (Mucin_bdg) and transmembrane (TMM) domains, and a disordered C-terminal structure.

We further assessed the most likely genomic determinants for the differences in UV tolerance by comparing the predicted viral structural proteins from the two studied strains. However, the amino acid sequences of polyhedrin (major protective protein), and many other major structural constituents of the OBs, were almost identical for both studied strains (Table 4). The most prominent genetic difference between the strains was related to the loss of the virus enhancin factor-1 (*vef-1*) gene in the *LdMNPV*-27/0 strain and a severe frameshift in the middle of the virus enhancin factor-2 (*vef-2*) gene in the *LdMNPV*-45/0 stain, which created a premature stop codon that presumably led to a loss of protein function and localization due to the absence of the transmembrane domain and a disordered structure in the C-terminal region (Fig 4).

**Table 4:** The inters-strain similarity of the major protein constituents of the OBs.

Protein name	Protein length		Identity,%	Coverage,%
	*LdMNPV*-45/0	*LdMNPV*-27/0		
Polyhedron envelope protein, pep (Ac131)	313	314	99.36	100
Polyhedrin (Ac8)	245	245	100	100
P10 protein (Ac137)	85	85	100	100
Envelope fusion protein, efp (Ac23)	676	676	99.41	100
Orf-73 protein (Ac68)	133	133	99.25	100
Major capsid protein, VP39	352	354	98.03	99
Viral enhancing factor 1 (enhancin), *vef-1*	783	gene loss	-	-
Viral enhancing factor 2 (enhancin), *vef-2*	514 (FS*)	788	58.17	38

* Frameshift with a premature stop codon

## Discussion

The present comparative study of two baculovirus strains isolated from different continents with significantly different initial potencies revealed that these strains possess significantly different tolerances to UV sunlight exposure. The strain isolated in North America (*LdMNPV*-45/0) lost its potency under sunlight treatment much faster than the Asian strain (*LdMNPV*-27/0). This occurred when larvae were infected with the same doses of two strains or with different doses to reach the same impact as 90% mortality. The effect of sunlight treatment on the time to host death after viral challenging was similar. Death was significantly delayed by 0.25 h of sunlight exposure for *LdMNPV*-45/0, while a significant delay was initiated only after 2 h of exposure for *LdMNPV*-27/0.

The loss of the potency of the virus was associated with direct effects of UV light on biopolymers [45–46]. UV light induced the rupture of DNA or led to the formation of pyrimidine dimers within the DNA chain or other photoproducts, such as pyrimidine photohydrates, thymine glycol, and DNA-DNA and DNA-protein binding agents [47–49]. UV was also able to cause indirect damage to DNA via the formation of reactive oxygen species (hydroperoxide, hydroxyl radical, singlet oxygen), which oxidized nucleotides and damaged the DNA chain [50–51]. For example, Ignoffo and Garcia [45] show that UV inactivation of baculoviruses was associated with the formation of reactive oxygen species using antioxidants as an adjuvant to prevent UV-induced inhibition of viral potency.

Recent work conducted on a mutant strain of *Adoxophyes orana* granulovirus showed that the size and shape of granules (occlusion bodies) are responsible for virus tolerance against UV [52]. To assess the possible impact of polyhedral morphology on the susceptibility to sunlight exposure observed in our study, we compared the shapes and sizes of OBs in the studied strains. However, no differences in the morphology of the OBs of the compared strains were found. The amino acid sequences of polyhedrin, as well as many other major structural constituents of OBs, were almost identical for both studied strains, except for (1) the deletion of the *vef-1* gene in *LdMNPV-*27/0 [34–35] and (2) a severe frameshift in the middle of the virus enhancin factor-2 (*vef-2*) gene in *LdMNPV*-45/0, which created a premature stop codon that presumably led to the loss of protein function and localization, due to the absence of the transmembrane domain and a disordered structure of the C-terminal portion. It is known that the products of the *vef* genes (*vef-1* and *vef-2*) of *LdMNPV* are structural proteins located in the ODV envelope, which are responsible for the high effectiveness of viral penetration in the host midgut tissue in the initial stages of baculoviral pathogenesis [53–55]. If a single deletion of the *vef-1* gene in *LdMNPV*-27/0 does decrease the potency of this virus strain compared to the *LdMNPV-*45/0 strain bearing this gene (as we suppose [35]), it is possible that the UV spectra of sunlight may partially inactivate the *vef-1* protein when the OBs of the *LdMNPV-*45/0 strain were exposed to UV. Several studies that have shown the effect of UV on viral proteins [41–43] indirectly support this assumption, but additional experiments are needed for direct confirmation of enhancin-1 inactivation. The significant decrease in time to death of the *LdMNPV-*45/0 strain relative to *LdMNPV*-27/0 under UV light may indirectly testify to the functioning of *vef-1* protein in the intensity of *LdMNPV* pathogenesis.

Prolonged sunlight treatment (2 h) increases the productivity of both viral strains during pathogeneses, but this occurred only when larvae were infected by LD_90_. The final number of progeny usually depends on host mass, which in turn depends on the duration of the feeding stage [56]. The two-hour sunlight treatment prolonged the time to death because of partial inactivation of the virus and a decrease in the live viral load concentration, which possibly led to an increased viral yield. We did not find the same effect of UV on virus productivity when comparing viral strains with the same concentration of OBs. Based on the inverse relationship between time to host death and virus productivity, we think that the low initial time to death recorded for the *LdMNPV*-27/0 strain at the virus concentration of 5x10^5^ OBs/larvae can explain the initial high productivity for that level of UV.

Thus, our study demonstrates that the effectiveness of the highly potent *LdMNPV* -45/0 strain ([34–35], the results of the current study) can be significantly reduced under natural UV irradiation in regions with high UV loading, which should be taken into account during pest management procedures. The role of the virus enhancin factor-1 in baculovirus tolerance to UV needs further study.

## Supporting information

S1 Dataset Initial dataset of all experiments described within the article. (XLS)

S1 Fig Effect of sunlight treatment on *Lymantria dispar* mortality dynamics. Dynamics of mortality of *Lymantria dispar* larvae challenged by *LdMNPV*-45/0 (dose 5x10^5^ OBs/larvae, a), *LdMNPV*-27/0 (dose 5x10^6^ OBs/larvae, b) and *LdMNPV*-27/0 (dose 5x10^5^ OBs/larvae, c) strains treated by sunlight. Statistical results of the time to death speed of mortality are given in Table 1.
(TIF)
