# Supplementary figures and images for: Comparison of tolerance to sunlight between spatially distant and genetically different strains of Lymantria dispar nucleopolyhedrovirus
Source: PLoS One. 2017 Dec 20;12(12):e0189992. doi: 10.1371/journal.pone.0189992 (PMC5738102; doi:10.1371/journal.pone.0189992)

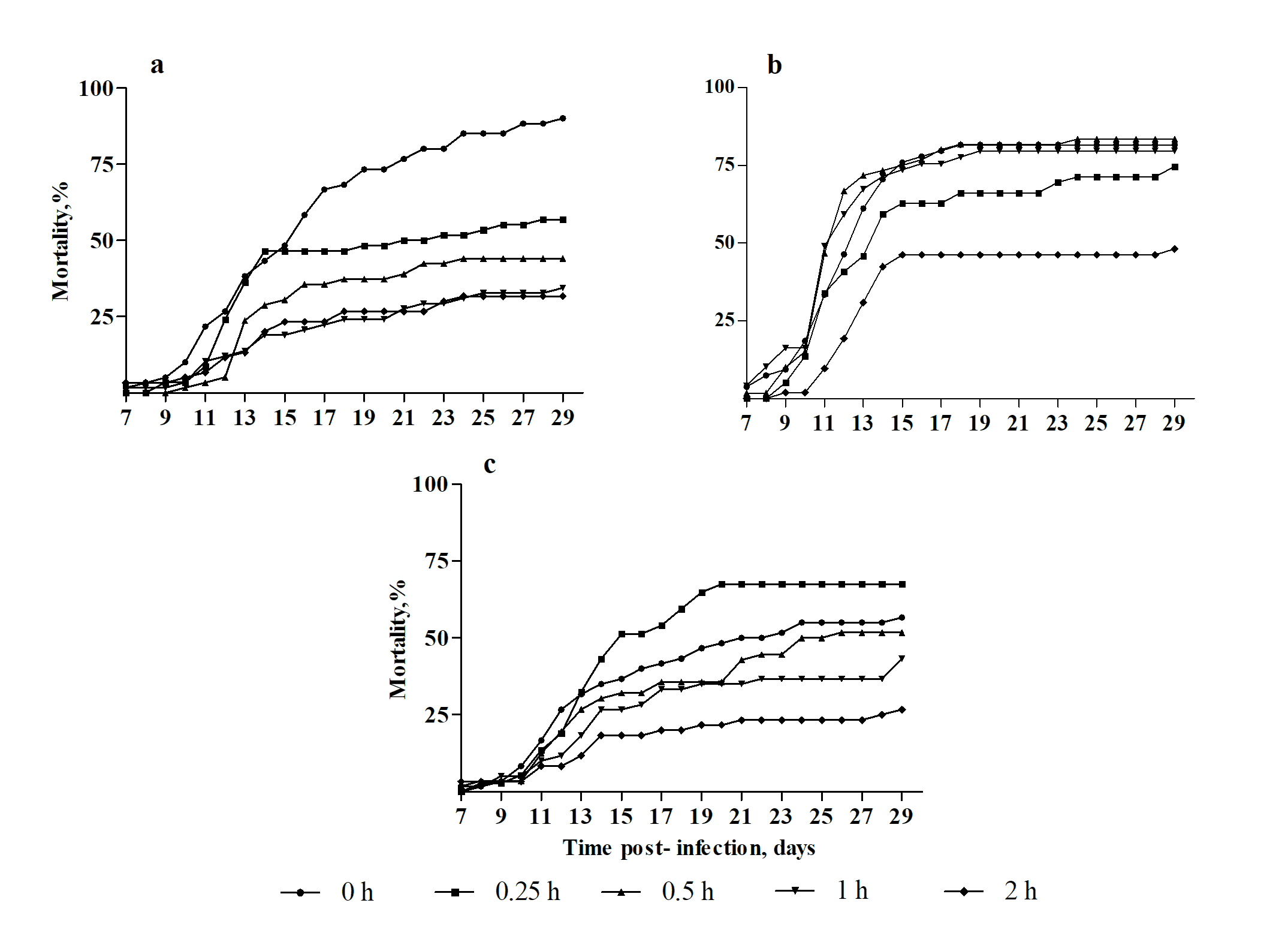

Supplement: S1 Fig — Dynamics of mortality of Lymantria dispar larvae challenged by LdMNPV-45/0 (dose 5x10^5^ OBs/larvae, a), LdMNPV-27/0 (dose 5x10^6^ OBs/larvae, b) and LdMNPV-27/0 (dose 5x10^5^ OBs/larvae, c) strains treated by sunlight. Statistical results of the time to death speed of mortality are given in Table 1. (TIF) [file pone.0189992.s002.tif]
